# Open fetal myeloschisis closure: pearls for uterine exposure and closure

**DOI:** 10.3171/2026.1.FOCVID25231

**Published:** 2026-04-01

**Authors:** David S. Hersh, Andrew J. Healy, Timothy M. Crombleholme

**Affiliations:** 1 Division of Neurosurgery, Connecticut Children’s, Hartford;; 2 Department of Neurosurgery, UConn School of Medicine, Farmington;; 3 Department of Pediatrics, UConn School of Medicine, Farmington;; 4 Connecticut Children’s Fetal Care Center, Connecticut Children’s, Hartford; and; 5 Division of Pediatric General, Thoracic, and Fetal Surgery, Connecticut Children’s, Hartford, Connecticut

**Keywords:** fetal, prenatal, myeloschisis, myelomeningocele, spina bifida, spinal, dysraphism, hysterotomy

## Abstract

Fetal closure of an open neural tube defect offers the potential to limit progressive neurological injury and improve long-term outcomes. Myeloschisis presents unique challenges due to the technical complexity of mobilizing the skin and achieving adequate soft tissue coverage, particularly in the sacral region. In this video, the authors demonstrate an open fetal myeloschisis closure in a fetus with an S1-level defect and associated grade 3 hindbrain herniation. The preoperative counseling process, surgical technique, and perioperative course are highlighted, with a focus on pearls for the exposure and closure of the uterus.

The video can be found here: https://stream.cadmore.media/r10.3171/2026.1.FOCVID25231

## Transcript

### 0:20 Introduction.

This video describes an open fetal closure of a neural tube defect—specifically, a patient with myeloschisis.

### 0:27 History and Diagnosis.

The patient was a 30-year-old female G5P2113 at 23 weeks and 6 days gestation. Prenatal imaging demonstrated an open neural tube defect at the S1 level. A CSF sac was not present underneath the neural placode, and the defect was therefore consistent with myeloschisis.^1,2^ The fetus was also noted to have grade 3 hindbrain herniation, moderate ventriculomegaly, appropriate movement at the hip, knee, and ankle, and no evidence of clubbed feet.

### 0:57 Preoperative Counseling.

We proceeded with multiphase, multidisciplinary counseling to discuss the management options, using a shared decision-making model.^3^ The team included Maternal-Fetal Medicine, Fetal Surgery, Pediatric Neurosurgery, and Neonatology. Three options were discussed, including interruption of the pregnancy, postnatal closure, and prenatal closure.

### 1:17 Prenatal Closure.

We reviewed that the primary goal of prenatal closure is to prevent progressive, secondary damage to the neural placode over the course of the pregnancy via chemical exposure to the amniotic fluid and physical trauma from the uterine wall. The landmark MOMS trial demonstrated that the benefits of prenatal closure include a lower rate of CSF diversion, improved ambulation, and in many cases, reversal of the fetus’s hindbrain herniation.^4^ However, these benefits must be weighed against the risks of maternal-fetal surgery, which include prematurity and its associated complications. In the MOMS trial, 46% of patients in the prenatal repair group delivered at or before 34 weeks, and 13% delivered before 30 weeks gestation. We also discussed a variety of obstetric complications including premature rupture of membranes, uterine dehiscence, oligohydramnios, chorio-amniotic separation, and placental abruption.

### 2:11 Prenatal Closure Options.

We then reviewed that a prenatal closure can be performed via an open hysterotomy, in which case a C-section is required prior to active labor for the current pregnancy and all future pregnancies due to the risk of uterine rupture. Alternatively, a minimally invasive approach called a fetoscopic closure involves laparoscopic instruments that are inserted through small ports into an exteriorized uterus.^5^ Vaginal delivery remains an option following a fetoscopic closure, but achieving an adequate closure is more technically challenging. In the current case, an open approach was recommended due to the nature of the open defect—in the setting of myeloschisis, skin mobilization and soft tissue coverage are often more difficult, and the closure is already more technically challenging, particularly in the sacral region.

Following a second phase of preoperative counseling that included meeting with a psychologist, the patient ultimately chose to proceed with an open fetal closure of the myeloschisis and was brought to the OR at 24 weeks and 6 days gestation.

### 3:08 Positioning.

The patient underwent placement of an epidural catheter followed by induction with selective intravenous anesthesia.^6^ An arterial line was placed and a Foley catheter was inserted. The patient was then placed in Allen stirrups, and all pressure points were well padded, with left uterine displacement using a rolled blanket.

### 3:27 Hysterotomy.

Following a timeout, a 14-cm transverse incision was made 4 fingerbreadths above the pubis and was carried down through the subcutaneous fat with electrocautery. Flaps were raised on the fascia up to the umbilicus and down to the pubis. The fascia was opened in the midline, a wound protector was placed, and the uterus was exteriorized. An inhalational agent, desflurane, was initiated to achieve uterine relaxation, and a version maneuver was performed to bring the fetus’s lumbosacral region to the site of the planned hysterotomy. The placental edge was mapped on the surface of the uterus at the fundus, and a hysterotomy was planned 2 fingerbreadths away. 4-0 PDS sutures were placed under ultrasound guidance to avoid the fetus and cord and create an avascular box. The hysterotomy was then performed with electrocautery, and Bainbridge clamps were used to compress the myometrium before coagulating and opening the hysterotomy for a length of 5 cm using a LigaSure device. A running 0 PDS was then used to close the myometrium on each side, obtain hemostasis, and prevent extension of the hysterotomy.

### 4:36 Myeloschisis Closure.

The Level 1 catheter was then placed in the amniotic cavity, and a cocktail of atropine, fentanyl, and vecuronium was injected into the left fetal thigh. We then proceeded with the myeloschisis closure. The neural placode was mobilized circumferentially using sharp dissection with microscissors. The skin was then undermined extensively. Bovie electrocautery with a Colorado needle was used to raise modified myofascial flaps. As described by Flanders et al.,^7^ this layer contains dura, muscle, fascia, and some cartilaginous bone inferiorly. After placing a small piece of cryopreserved decellularized human umbilical cord matrix allograft over the released placode to provide a scaffold for tissue repair and regeneration,^8,9^ the modified myofascial flaps were then rotated over the placode, with dura lining the deep aspect of the flaps, and were closed with a running 5-0 PDS. The skin was then reapproximated with a running 5-0 PDS. A primary watertight closure was achieved, and neither relaxing incisions nor a skin patch were required.

### 5:37 Closing.

We then turned our attention to the hysterotomy closure. Full-thickness interrupted 0 PDS retention sutures were placed ~2 cm from the edge of the hysterotomy, incorporating the myometrium and membranes every 3 cm. A 0 PDS was then run from one corner to the other to close the hysterotomy. The Level 1 was used to normalize the amniotic fluid with a maximum vertical pocket of 3.5 cm, and 2 g of nafcillin were administered via the Level 1 catheter before it was removed. The running stitch was then tied, followed by the interrupted 0 PDS sutures. A third layer of 0 PDS sutures were placed in a U-shaped fashion to imbricate the closure with an additional myometrial layer, providing serosa to serosa closure.^10^ The wound protector was removed, and a layer of omentum was tacked into place over the hysterotomy site. The uterus was then returned to the abdomen. The wound was then copiously irrigated with warm normal saline, and the midline fascial incision was closed with a running 0 Vicryl suture. The flaps were reapproximated with a series of 2-0 Vicryls, anchoring the flaps to the fascia. A second layer of 2-0 Vicryls were placed subcutaneously, and the skin was closed with a running 4-0 Monocryl. The closure was reinforced with Dermabond. A transvaginal ultrasound revealed no change in the cervical length.

### 7:28 Fetal Echocardiography.

Fetal echocardiography was performed continuously during the procedure, revealing a fetal heart rate ranging from 126 to 132 beats per minute. In addition, AV valve competence and ductus arteriosus flow velocities did not change during the procedure.

### 7:44 Postoperative Course.

The patient was then taken out of the Allen stirrups, extubated, and transported in stable condition to the labor and delivery unit. She was ultimately discharged on postoperative day number 3. Her pregnancy was subsequently complicated by preterm premature rupture of membranes and preterm labor at 27 weeks and 1 day, and she underwent an unplanned cesarean section. The newborn period was complicated by respiratory distress syndrome treated with nasal cannula CPAP, and the infant was ultimately weaned to room air. There were no significant cardiovascular concerns nor necrotizing enterocolitis. Although there were initial concerns for sepsis based on maternal presentation, blood cultures were ultimately negative and prophylactic treatment was discontinued. No evidence of retinopathy of prematurity was noted on exams, which took place throughout the admission. However, continued follow-up will be performed as an outpatient. As of 7 weeks postdelivery, head ultrasounds have demonstrated stable moderate ventriculomegaly with no evidence of acute intracranial hemorrhage. She remains clinically stable, has a stable head circumference at the 41st percentile with a soft fontanelle, exhibits spontaneous movement of all muscle groups in the lower extremities, and has a well healed incision.

### 8:57 Pearls for the Exposure and Closure.

We would like to highlight several features of our surgical technique, with particular attention to the exposure and closure of the uterus. To decrease fetal exposure to high levels of a volatile anesthetic agent, supplemental intravenous anesthesia is initiated immediately after anesthesia induction until uterine relaxation is required, at which point desflurane is initiated.^6^ This avoids desflurane exposure while additional vascular access is obtained, an ultrasound assessment is performed, the bladder is catheterized, and the patient is prepped and draped. Ultimately, when desflurane is initiated for uterine relaxation, we have observed a 2% incidence of idiopathic fetal bradycardia, with a heart rate in the range of 100–110. This bradycardic effect appears to be both dose-dependent and reversible. Though umbilical cord compression must be maintained within the differential diagnosis, and a fetal echocardiogram must be obtained, the bradycardia often self-resolves after turning off the inhalational agent. Finally, the modified hysterotomy closure described here incorporates a third imbricating layer, resulting in serosal-to-serosal apposition. This modified technique has nearly eliminated uterine dehiscence and has significantly reduced the rate of thinning of the uterine scar.^10^

## Disclosures

The authors report no conflict of interest concerning the materials or methods used in this study or the findings specified in this publication.

## Author Contributions

Primary surgeon: Hersh, Crombleholme. Assistant surgeon: Healy. Editing and drafting the video and abstract: all authors. Critically revising the work: Hersh, Crombleholme. Reviewed submitted version of the work: Hersh, Crombleholme. Approved the final version of the work on behalf of all authors: Hersh.

## Supplemental Information

### Patient Informed Consent

The necessary patient informed consent was obtained in this study.
